# *cis*-Dichloridobis(quinoline-κ*N*)­platinum(II) nitro­methane monosolvate

**DOI:** 10.1107/S1600536812012469

**Published:** 2012-03-28

**Authors:** Kwang Ha

**Affiliations:** a School of Applied Chemical Engineering, Research Institute of Catalysis, Chonnam National University, Gwangju 500-757, Republic of Korea

## Abstract

In the title compound, [PtCl_2_(C_9_H_7_N)_2_]·CH_3_NO_2_, the Pt^II^ cation is four-coordinated in an essentially square-planar environment by two N atoms from two quinoline ligands and two Cl^−^ anions. One of the nearly planar quinoline ligands [maximum deviations = 0.042 (6) and 0.018 (7) Å] is almost perpendicular to the PtCl_2_N_2_ unit [maximum deviation = 0.024 (3) Å], making a dihedral angle of 89.6 (1)°, whereas the other is slightly inclined to the central plane with a dihedral angle of 74.1 (1)°. The dihedral angle between the quinoline ligands is 88.3 (2)°. In the crystal, each solvent mol­ecule is linked to the metal complex by weak inter­molecular C—H⋯O hydrogen bonds.

## Related literature

For the crystal structure of *cis*-[PtCl_2_(quinoline)_2_]·0.25DMF (DMF = *N*,*N*-dimethyl­formamide), see: Davies *et al.* (2001 ▶). For the crystal structure of the related Pd^II^ complex *trans*-[PdCl_2_(quinoline)_2_], see: Ha (2012 ▶).
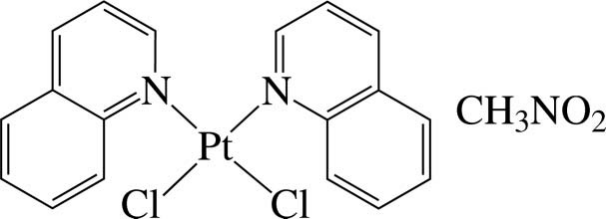


## Experimental

### 

#### Crystal data

[PtCl_2_(C_9_H_7_N)_2_]·CH_3_NO_2_
*M* *_r_* = 585.35
Triclinic, 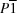

*a* = 9.6204 (5) Å
*b* = 10.3698 (5) Å
*c* = 11.6946 (6) Å
α = 104.244 (1)°
β = 101.913 (1)°
γ = 113.834 (1)°
*V* = 970.87 (8) Å^3^
*Z* = 2
Mo *K*α radiation
μ = 7.52 mm^−1^
*T* = 200 K
0.25 × 0.19 × 0.13 mm

#### Data collection

Bruker SMART 1000 CCD diffractometer
Absorption correction: multi-scan (*SADABS*; Bruker, 2000 ▶) *T*_min_ = 0.754, *T*_max_ = 1.000
6035 measured reflections
3707 independent reflections
3342 reflections with *I* > 2σ(*I*)
*R*_int_ = 0.021

#### Refinement

*R*[*F* ^2^ > 2σ(*F* ^2^)] = 0.030
*wR*(*F* ^2^) = 0.084
*S* = 1.24
3707 reflections
245 parameters
H-atom parameters constrained
Δρ_max_ = 2.69 e Å^−3^
Δρ_min_ = −1.38 e Å^−3^

### 

Data collection: *SMART* (Bruker, 2000 ▶); cell refinement: *SAINT* (Bruker, 2000 ▶); data reduction: *SAINT*; program(s) used to solve structure: *SHELXS97* (Sheldrick, 2008 ▶); program(s) used to refine structure: *SHELXL97* (Sheldrick, 2008 ▶); molecular graphics: *ORTEP-3* (Farrugia, 1997 ▶) and *PLATON* (Spek, 2009 ▶); software used to prepare material for publication: *SHELXL97*.

## Supplementary Material

Crystal structure: contains datablock(s) global, I. DOI: 10.1107/S1600536812012469/xu5489sup1.cif

Structure factors: contains datablock(s) I. DOI: 10.1107/S1600536812012469/xu5489Isup2.hkl

Additional supplementary materials:  crystallographic information; 3D view; checkCIF report

## Figures and Tables

**Table:** 

Pt1—N1	2.045 (6)
Pt1—N2	2.045 (6)
Pt1—Cl1	2.2881 (18)
Pt1—Cl2	2.3019 (19)

**Table:** 

N2—Pt1—N1	90.3 (2)
Cl1—Pt1—Cl2	91.99 (7)

**Table 2:** Hydrogen-bond geometry (Å, °)

*D*—H⋯*A*	*D*—H	H⋯*A*	*D*⋯*A*	*D*—H⋯*A*
C16—H16⋯O2^i^	0.95	2.59	3.323 (13)	134

Symmetry code: (i) 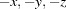.

**Fig. 1.**
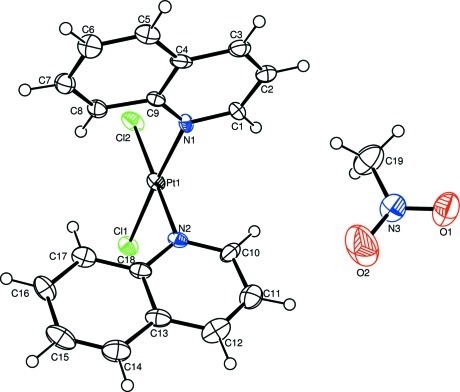
A structure detail of the title compound, with atom numbering. Displacement ellipsoids are drawn at the 40% probability level for non-H atoms.

**Fig. 2.**
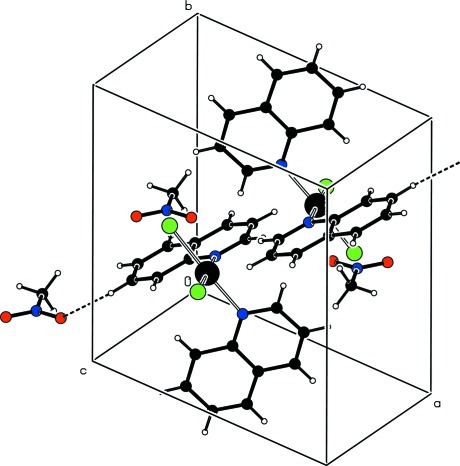
A view of the unit-cell contents of the title compound. Intermolecular C—H···O hydrogen-bond interactions are drawn with dashed lines.

## supplementary crystallographic information

### Comment

The asymmetric unit of the title compound, [PtCl_2_(quinoline)_2_].CH_3_NO_2_, contains a neutral Pt^II^ complex and a nitromethane solvent molecule (Fig. 1). In the complex, the Pt^II^ ion is four-coordinated in an essentially square-planar environment by two N atoms from two quinoline ligands and two Cl^-^ anions (Table 1). The Cl atoms are in *cis* conformation with respect to each other, like in the analogous Pt^II^ complex [PtCl_2_(quinoline)_2_].0.25DMF (DMF = *N,N*-dimethylformamide) (Davies *et al.*, 2001). By contrast, in the related Pd^II^ complex [PdCl_2_(quinoline)_2_], the Cl atoms are in *trans* conformation (Ha, 2012).

One of the nearly planar quinoline ligands [maximum deviations = 0.042 (6) Å and 0.018 (7) Å] is almost perpendicular to the PtCl_2_N_2_ unit [maximum deviation = 0.024 (3) Å], making dihedral angle of 89.6 (1)°, whereas the other is slightly inclined to the unit plane with a dihedral angle of 74.1 (1)°. The dihedral angle between the quinoline ligands is 88.3 (2)°. In the crystal, each solvent molecule is linked to the complex by intermolecular C—H···O hydrogen bonds (Fig. 2 and Table 2). Moreover, the complex molecules display numerous intermolecular π-π interactions between adjacent six-membered rings, the shortest ring centroid-centroid distance being 3.617 (5) Å.

### Experimental

To a solution of K_2_PtCl_4_ (0.2074 g, 0.500 mmol) in H_2_O (20 ml) was added quinoline (0.1304 g, 1.010 mmol), and refluxed for 3 h. The formed precipitate was separated by filtration, washed with H_2_O and EtOH, and dried at 50°C, to give a yellow powder (0.1761 g). Crystals suitable for X-ray analysis were obtained by slow evaporation from a CH_3_NO_2_ solution at room temperature.

### Refinement

H atoms were positioned geometrically and allowed to ride on their respective parent atoms: C—H = 0.95 Å (CH) or 0.98 Å (CH_3_) with *U*_iso_(H) = 1.2*U*_eq_(C) or 1.5*U*_eq_(methyl C). The highest peak (2.69 e Å^-3^) and the deepest hole (-1.38 e Å^-3^) in the difference Fourier map are located 0.86 Å and 0.75 Å, respectively, from the Pt1 atom.

### Crystal data

**Table:**

[PtCl_2_(C_9_H_7_N)_2_]·CH_3_NO_2_	*Z* = 2
*M**_r_* = 585.35	*F*(000) = 560
Triclinic, *P*1	*D*_x_ = 2.002 Mg m^−^^3^
Hall symbol: -P 1	Mo *K*α radiation, λ = 0.71073 Å
*a* = 9.6204 (5) Å	Cell parameters from 4362 reflections
*b* = 10.3698 (5) Å	θ = 2.4–26.0°
*c* = 11.6946 (6) Å	µ = 7.52 mm^−^^1^
α = 104.244 (1)°	*T* = 200 K
β = 101.913 (1)°	Block, yellow
γ = 113.834 (1)°	0.25 × 0.19 × 0.13 mm
*V* = 970.87 (8) Å^3^	

### Data collection

**Table:**

Bruker SMART 1000 CCD diffractometer	3707 independent reflections
Radiation source: fine-focus sealed tube	3342 reflections with *I* > 2σ(*I*)
Graphite monochromator	*R*_int_ = 0.021
φ and ω scans	θ_max_ = 26.0°, θ_min_ = 1.9°
Absorption correction: multi-scan (*SADABS*; Bruker, 2000)	*h* = −9→11
*T*_min_ = 0.754, *T*_max_ = 1.000	*k* = −12→12
6035 measured reflections	*l* = −14→14

### Refinement

**Table:**

Refinement on *F*^2^	Primary atom site location: structure-invariant direct methods
Least-squares matrix: full	Secondary atom site location: difference Fourier map
*R*[*F*^2^ > 2σ(*F*^2^)] = 0.030	Hydrogen site location: inferred from neighbouring sites
*wR*(*F*^2^) = 0.084	H-atom parameters constrained
*S* = 1.24	*w* = 1/[σ^2^(*F*_o_^2^) + (0.*P*)^2^ + 7.8267*P*] where *P* = (*F*_o_^2^ + 2*F*_c_^2^)/3
3707 reflections	(Δ/σ)_max_ < 0.001
245 parameters	Δρ_max_ = 2.69 e Å^−^^3^
0 restraints	Δρ_min_ = −1.38 e Å^−^^3^

### Special details

**Table:**

Geometry. All e.s.d.'s (except the e.s.d. in the dihedral angle between two l.s. planes) are estimated using the full covariance matrix. The cell e.s.d.'s are taken into account individually in the estimation of e.s.d.'s in distances, angles and torsion angles; correlations between e.s.d.'s in cell parameters are only used when they are defined by crystal symmetry. An approximate (isotropic) treatment of cell e.s.d.'s is used for estimating e.s.d.'s involving l.s. planes.
Refinement. Refinement of *F*^2^ against ALL reflections. The weighted *R*-factor *wR* and goodness of fit *S* are based on *F*^2^, conventional *R*-factors *R* are based on *F*, with *F* set to zero for negative *F*^2^. The threshold expression of *F*^2^ > σ(*F*^2^) is used only for calculating *R*-factors(gt) *etc*. and is not relevant to the choice of reflections for refinement. *R*-factors based on *F*^2^ are statistically about twice as large as those based on *F*, and *R*- factors based on ALL data will be even larger.

### Fractional atomic coordinates and isotropic or equivalent isotropic displacement parameters (Å^2^)

**Table:**

	*x*	*y*	*z*	*U*_iso_*/*U*_eq_	
Pt1	0.24674 (4)	0.27007 (3)	0.45574 (3)	0.02575 (10)	
Cl1	0.0985 (2)	0.3934 (2)	0.48336 (18)	0.0326 (4)	
Cl2	0.2977 (3)	0.2747 (3)	0.65828 (18)	0.0395 (5)	
N1	0.3854 (7)	0.1676 (7)	0.4262 (6)	0.0245 (13)	
N2	0.2011 (7)	0.2682 (7)	0.2765 (6)	0.0252 (13)	
C1	0.5394 (9)	0.2527 (9)	0.4458 (7)	0.0284 (16)	
H1	0.5840	0.3595	0.4829	0.034*	
C2	0.6416 (10)	0.1950 (9)	0.4153 (7)	0.0327 (18)	
H2	0.7528	0.2614	0.4333	0.039*	
C3	0.5792 (10)	0.0430 (9)	0.3596 (7)	0.0310 (17)	
H3	0.6456	0.0013	0.3368	0.037*	
C4	0.4121 (9)	−0.0533 (9)	0.3356 (7)	0.0279 (16)	
C5	0.3400 (10)	−0.2136 (9)	0.2791 (7)	0.0345 (18)	
H5	0.4021	−0.2590	0.2533	0.041*	
C6	0.1834 (10)	−0.3010 (9)	0.2622 (7)	0.0358 (19)	
H6	0.1349	−0.4078	0.2213	0.043*	
C7	0.0899 (10)	−0.2368 (9)	0.3042 (7)	0.0331 (18)	
H7	−0.0189	−0.3013	0.2944	0.040*	
C8	0.1541 (9)	−0.0833 (8)	0.3586 (6)	0.0274 (16)	
H8	0.0909	−0.0407	0.3869	0.033*	
C9	0.3187 (8)	0.0128 (8)	0.3725 (6)	0.0218 (14)	
C10	0.3191 (11)	0.3682 (9)	0.2522 (8)	0.0331 (18)	
H10	0.4228	0.4291	0.3160	0.040*	
C11	0.2975 (11)	0.3873 (9)	0.1381 (8)	0.041 (2)	
H11	0.3854	0.4592	0.1243	0.049*	
C12	0.1504 (12)	0.3031 (9)	0.0463 (8)	0.044 (2)	
H12	0.1338	0.3173	−0.0316	0.053*	
C13	0.0218 (10)	0.1942 (9)	0.0665 (7)	0.0333 (18)	
C14	−0.1344 (12)	0.1003 (11)	−0.0233 (8)	0.044 (2)	
H14	−0.1561	0.1111	−0.1024	0.052*	
C15	−0.2533 (12)	−0.0033 (11)	−0.0024 (8)	0.049 (2)	
H15	−0.3571	−0.0651	−0.0660	0.059*	
C16	−0.2242 (10)	−0.0207 (10)	0.1153 (8)	0.040 (2)	
H16	−0.3084	−0.0954	0.1300	0.048*	
C17	−0.0761 (10)	0.0692 (9)	0.2076 (7)	0.0343 (18)	
H17	−0.0582	0.0585	0.2869	0.041*	
C18	0.0508 (9)	0.1783 (8)	0.1850 (7)	0.0275 (16)	
O1	0.8350 (9)	0.4264 (8)	0.0474 (7)	0.067 (2)	
O2	0.5859 (11)	0.3279 (13)	0.0137 (9)	0.112 (4)	
N3	0.7193 (10)	0.3685 (8)	0.0788 (7)	0.0438 (18)	
C19	0.7469 (14)	0.3484 (11)	0.1983 (9)	0.061 (3)	
H19A	0.7237	0.4160	0.2566	0.091*	
H19B	0.8600	0.3726	0.2334	0.091*	
H19C	0.6756	0.2430	0.1857	0.091*	

### Atomic displacement parameters (Å^2^)

**Table:**

	*U*^11^	*U*^22^	*U*^33^	*U*^12^	*U*^13^	*U*^23^
Pt1	0.02316 (16)	0.02821 (16)	0.02450 (16)	0.01381 (13)	0.00675 (12)	0.00619 (12)
Cl1	0.0309 (10)	0.0313 (10)	0.0369 (10)	0.0193 (9)	0.0113 (8)	0.0075 (8)
Cl2	0.0361 (11)	0.0639 (14)	0.0288 (10)	0.0318 (11)	0.0133 (9)	0.0177 (10)
N1	0.020 (3)	0.033 (3)	0.029 (3)	0.014 (3)	0.016 (3)	0.015 (3)
N2	0.026 (3)	0.025 (3)	0.028 (3)	0.018 (3)	0.007 (3)	0.008 (3)
C1	0.017 (4)	0.032 (4)	0.033 (4)	0.010 (3)	0.005 (3)	0.014 (3)
C2	0.032 (4)	0.038 (5)	0.032 (4)	0.017 (4)	0.008 (4)	0.021 (4)
C3	0.039 (5)	0.042 (5)	0.030 (4)	0.027 (4)	0.017 (4)	0.022 (4)
C4	0.032 (4)	0.040 (4)	0.028 (4)	0.027 (4)	0.012 (3)	0.020 (3)
C5	0.037 (5)	0.038 (5)	0.035 (4)	0.025 (4)	0.011 (4)	0.013 (4)
C6	0.038 (5)	0.029 (4)	0.031 (4)	0.015 (4)	0.006 (4)	0.003 (3)
C7	0.028 (4)	0.026 (4)	0.039 (5)	0.012 (3)	0.006 (4)	0.010 (3)
C8	0.025 (4)	0.031 (4)	0.022 (4)	0.012 (3)	0.003 (3)	0.009 (3)
C9	0.020 (4)	0.026 (4)	0.018 (3)	0.012 (3)	0.002 (3)	0.007 (3)
C10	0.044 (5)	0.033 (4)	0.039 (5)	0.023 (4)	0.024 (4)	0.024 (4)
C11	0.046 (5)	0.028 (4)	0.047 (5)	0.016 (4)	0.017 (4)	0.013 (4)
C12	0.069 (7)	0.033 (5)	0.029 (4)	0.025 (5)	0.017 (5)	0.008 (4)
C13	0.040 (5)	0.032 (4)	0.025 (4)	0.022 (4)	0.001 (4)	0.005 (3)
C14	0.052 (6)	0.053 (6)	0.030 (5)	0.031 (5)	0.010 (4)	0.014 (4)
C15	0.039 (5)	0.061 (6)	0.031 (5)	0.026 (5)	−0.005 (4)	0.002 (4)
C16	0.026 (4)	0.044 (5)	0.034 (5)	0.011 (4)	0.002 (4)	0.003 (4)
C17	0.039 (5)	0.040 (5)	0.023 (4)	0.023 (4)	0.009 (4)	0.007 (3)
C18	0.031 (4)	0.027 (4)	0.026 (4)	0.019 (3)	0.005 (3)	0.007 (3)
O1	0.062 (5)	0.061 (5)	0.064 (5)	0.013 (4)	0.025 (4)	0.028 (4)
O2	0.051 (6)	0.168 (10)	0.072 (6)	0.023 (6)	0.001 (5)	0.043 (7)
N3	0.042 (5)	0.034 (4)	0.027 (4)	0.004 (4)	0.000 (3)	0.001 (3)
C19	0.071 (8)	0.049 (6)	0.041 (6)	0.013 (6)	0.010 (5)	0.017 (5)

### Geometric parameters (Å, º)

**Table:**

Pt1—N1	2.045 (6)	C8—H8	0.9500
Pt1—N2	2.045 (6)	C10—C11	1.383 (11)
Pt1—Cl1	2.2881 (18)	C10—H10	0.9500
Pt1—Cl2	2.3019 (19)	C11—C12	1.353 (13)
N1—C1	1.315 (9)	C11—H11	0.9500
N1—C9	1.375 (9)	C12—C13	1.409 (12)
N2—C10	1.331 (10)	C12—H12	0.9500
N2—C18	1.373 (9)	C13—C14	1.404 (12)
C1—C2	1.403 (11)	C13—C18	1.420 (10)
C1—H1	0.9500	C14—C15	1.332 (13)
C2—C3	1.355 (11)	C14—H14	0.9500
C2—H2	0.9500	C15—C16	1.418 (12)
C3—C4	1.428 (11)	C15—H15	0.9500
C3—H3	0.9500	C16—C17	1.364 (11)
C4—C9	1.405 (10)	C16—H16	0.9500
C4—C5	1.420 (11)	C17—C18	1.416 (11)
C5—C6	1.348 (12)	C17—H17	0.9500
C5—H5	0.9500	O1—N3	1.213 (10)
C6—C7	1.413 (11)	O2—N3	1.185 (11)
C6—H6	0.9500	N3—C19	1.450 (11)
C7—C8	1.366 (10)	C19—H19A	0.9800
C7—H7	0.9500	C19—H19B	0.9800
C8—C9	1.438 (10)	C19—H19C	0.9800
			
N2—Pt1—N1	90.3 (2)	C4—C9—C8	119.4 (7)
N2—Pt1—Cl1	87.40 (17)	N2—C10—C11	122.9 (8)
N1—Pt1—Cl1	176.81 (17)	N2—C10—H10	118.5
N2—Pt1—Cl2	179.33 (17)	C11—C10—H10	118.5
N1—Pt1—Cl2	90.30 (17)	C12—C11—C10	119.6 (8)
Cl1—Pt1—Cl2	91.99 (7)	C12—C11—H11	120.2
C1—N1—C9	118.8 (6)	C10—C11—H11	120.2
C1—N1—Pt1	118.8 (5)	C11—C12—C13	119.8 (8)
C9—N1—Pt1	121.9 (5)	C11—C12—H12	120.1
C10—N2—C18	119.4 (7)	C13—C12—H12	120.1
C10—N2—Pt1	117.8 (5)	C14—C13—C12	124.0 (8)
C18—N2—Pt1	122.5 (5)	C14—C13—C18	117.6 (8)
N1—C1—C2	123.9 (7)	C12—C13—C18	118.3 (7)
N1—C1—H1	118.1	C15—C14—C13	122.9 (8)
C2—C1—H1	118.1	C15—C14—H14	118.6
C3—C2—C1	118.9 (8)	C13—C14—H14	118.6
C3—C2—H2	120.5	C14—C15—C16	119.6 (8)
C1—C2—H2	120.5	C14—C15—H15	120.2
C2—C3—C4	118.9 (7)	C16—C15—H15	120.2
C2—C3—H3	120.5	C17—C16—C15	120.4 (9)
C4—C3—H3	120.5	C17—C16—H16	119.8
C9—C4—C5	119.6 (7)	C15—C16—H16	119.8
C9—C4—C3	118.7 (7)	C16—C17—C18	120.0 (8)
C5—C4—C3	121.7 (7)	C16—C17—H17	120.0
C6—C5—C4	119.9 (7)	C18—C17—H17	120.0
C6—C5—H5	120.0	N2—C18—C17	120.6 (7)
C4—C5—H5	120.0	N2—C18—C13	119.9 (7)
C5—C6—C7	121.2 (7)	C17—C18—C13	119.4 (7)
C5—C6—H6	119.4	O2—N3—O1	121.4 (9)
C7—C6—H6	119.4	O2—N3—C19	120.1 (9)
C8—C7—C6	120.8 (8)	O1—N3—C19	118.5 (8)
C8—C7—H7	119.6	N3—C19—H19A	109.5
C6—C7—H7	119.6	N3—C19—H19B	109.5
C7—C8—C9	119.0 (7)	H19A—C19—H19B	109.5
C7—C8—H8	120.5	N3—C19—H19C	109.5
C9—C8—H8	120.5	H19A—C19—H19C	109.5
N1—C9—C4	120.6 (7)	H19B—C19—H19C	109.5
N1—C9—C8	120.0 (6)		
			
N2—Pt1—N1—C1	86.5 (6)	C5—C4—C9—C8	3.3 (10)
Cl2—Pt1—N1—C1	−93.2 (5)	C3—C4—C9—C8	−174.8 (6)
N2—Pt1—N1—C9	−85.6 (5)	C7—C8—C9—N1	179.6 (7)
Cl2—Pt1—N1—C9	94.7 (5)	C7—C8—C9—C4	−3.1 (10)
N1—Pt1—N2—C10	−75.1 (5)	C18—N2—C10—C11	0.5 (11)
Cl1—Pt1—N2—C10	102.7 (5)	Pt1—N2—C10—C11	−173.6 (6)
N1—Pt1—N2—C18	111.0 (5)	N2—C10—C11—C12	0.8 (12)
Cl1—Pt1—N2—C18	−71.3 (5)	C10—C11—C12—C13	−1.6 (13)
C9—N1—C1—C2	−0.1 (11)	C11—C12—C13—C14	−179.2 (8)
Pt1—N1—C1—C2	−172.4 (6)	C11—C12—C13—C18	1.1 (12)
N1—C1—C2—C3	1.6 (11)	C12—C13—C14—C15	179.1 (9)
C1—C2—C3—C4	−0.9 (11)	C18—C13—C14—C15	−1.2 (13)
C2—C3—C4—C9	−1.0 (10)	C13—C14—C15—C16	0.5 (14)
C2—C3—C4—C5	−179.1 (7)	C14—C15—C16—C17	0.9 (14)
C9—C4—C5—C6	−0.5 (11)	C15—C16—C17—C18	−1.5 (13)
C3—C4—C5—C6	177.6 (7)	C10—N2—C18—C17	178.9 (7)
C4—C5—C6—C7	−2.5 (12)	Pt1—N2—C18—C17	−7.2 (9)
C5—C6—C7—C8	2.7 (12)	C10—N2—C18—C13	−1.0 (10)
C6—C7—C8—C9	0.2 (11)	Pt1—N2—C18—C13	172.8 (5)
C1—N1—C9—C4	−1.9 (10)	C16—C17—C18—N2	−179.2 (7)
Pt1—N1—C9—C4	170.2 (5)	C16—C17—C18—C13	0.7 (11)
C1—N1—C9—C8	175.3 (6)	C14—C13—C18—N2	−179.5 (7)
Pt1—N1—C9—C8	−12.6 (9)	C12—C13—C18—N2	0.2 (11)
C5—C4—C9—N1	−179.4 (7)	C14—C13—C18—C17	0.6 (11)
C3—C4—C9—N1	2.5 (10)	C12—C13—C18—C17	−179.7 (7)

### Hydrogen-bond geometry (Å, º)

**Table:**

*D*—H···*A*	*D*—H	H···*A*	*D*···*A*	*D*—H···*A*
C16—H16···O2^i^	0.95	2.59	3.323 (13)	134

Symmetry code: (i) −*x*, −*y*, −*z*.
